# Root-Zone Warming Differently Benefits Mature and Newly Unfolded Leaves of *Cucumis sativus* L. Seedlings under Sub-Optimal Temperature Stress

**DOI:** 10.1371/journal.pone.0155298

**Published:** 2016-05-06

**Authors:** Xiaozhuo Wang, Weihua Zhang, Yanxiu Miao, Lihong Gao

**Affiliations:** Beijing Key Laboratory of Growth and Developmental Regulation for Protected Vegetable Crops, China Agricultural University, Beijing, China; National Research Council of Italy, ITALY

## Abstract

Sub-optimal temperature extensively suppresses crop growth during cool-season greenhouse production. Root-zone (RZ) warming is considered an economical option to alleviate crop growth reduction. In this study we cultivated cucumber seedlings in nutrient solution under different air-RZ temperature treatments to investigate the effects of RZ warming on seedling growth- and photosynthesis-related parameters in leaves. The air-RZ temperature treatments included sub-optimal RZ temperature 13°C and sub-optimal air temperature 20/12°C (day/night) (S13), RZ warming at 19°C and sub-optimal air temperature (S19), and RZ warming at 19°C and optimal air temperature 26/18°C (day/night) (O19). In addition, for each air-RZ temperature treatment, half of the seedlings were also treated with 2% (*m/m*) polyethylene glycol (PEG) dissolved in nutrient solution to distinguish the effect of root-sourced water supply from RZ temperature. At the whole-plant level, S19 significantly increased the relative growth rate (RGR) by approximately 18% compared with S13, although the increase was less than in O19 (50%) due to delayed leaf emergence. S19 alleviated both diffusive and metabolic limitation of photosynthesis in mature leaves compared with S13, resulting in a photosynthetic rate similar to that in O19 leaves. In newly unfolded leaves, S19 significantly promoted leaf area expansion and alleviated stomatal limitation of photosynthesis compared with S13. PEG addition had a limited influence on RGR and leaf photosynthesis, but significantly suppressed new leaf expansion. Thus, our results indicate that under sub-optimal temperature conditions, RZ warming promotes cucumber seedling growth by differently benefiting mature and newly unfolded leaves. In addition, RZ warming enhanced root-sourced water supply, mainly promoting new leaf expansion, rather than photosynthesis.

## Introduction

Low temperature is a prominent limiting factor for horticultural crop production in temperate regions, particularly during cool-season cultivation. Although chilling conditions can be largely avoided in heated or solar greenhouses, insufficient heating due to high fuel costs and/or long-term environmental considerations frequently result in sub-optimal temperature conditions, which usually persist for a long period [1,2]. Sub-optimal temperature stress usually leads to reduced vegetative growth, which may adversely affect yield and fruit quality [1]. Thus, to overcome the low-temperature imposed decrease of green-house plant production and to reduce energy consumption, environment control techniques that decrease heating costs, or raise crop resistance to unfavorable temperatures are required.

Root-zone (RZ) temperature regulation is a promising approach for maintaining plant health under sub-optimal temperatures [3,4], mainly because when compared with the aerial part, the RZ has a higher specific heat capacity and is more easily controlled [3,5]. Most importantly, a suitable RZ temperature is indispensable for plant health. Many studies have focused on the negative impacts of unfavorable RZ temperature on plant growth and physiological activities [6–11]. Improved production by applying root-warming/cooling methods has been reported in some field experiments [3–4,12–13]. However, most of the former studies were conducted under optimum air temperature conditions, and the specific effects of RZ temperature under unfavorable shoot temperature remain unclear.

One of the most remarkable effects induced by changing root temperature is the alteration of water uptake, which may have far-reaching implications. A number of studies have reported that a RZ temperature below the optimum decreases the water uptake ability of the root [7,14–17]. In addition, it has been demonstrated that decreased root-sourced water supply is strongly related to reduced leaf growth [10] and stomatal conductance [14,18], which will negatively affect the overall assimilation capability of a plant. However, it is still unclear how water uptake regulates plant growth under changes in root-zone temperature, because no study has investigated water uptake separate from other effects induced by root temperature conditions.

Despite the potential benefits of a so-called “non-stressful” RZ temperature for plants, negative effects may also occur. For example, Suzuki et al. [19] observed severe, visible leaf damage when chilled rice seedlings were provided with root warming at 25°C. The chilling injury was related to the blockage of photosynthetic electron transport, which was induced by the warm RZ temperature [20]. Soto et al. and Paredes et al. recently reported similar results [21,22]. Hao et al. observed that the activities of photosystem II (PSII) and antioxidant enzymes declined when heated peach seedlings were provided a non-stressful root temperature [23]. Because the interaction between roots and shoots under stressful conditions is complex and still unclear, the identification of a strategy for optimal environmental control was not as simple as expected in these studies. Therefore, further investigation of RZ temperature regulation is required to develop a deeper understanding of plant root-shoot communication and proper strategies for environmental control under non-optimal or stressful temperatures.

The objective of this study was to investigate the effects of RZ warming at a sub-optimal air temperature on growth characteristics and photosynthesis-related parameters in cucumber (*Cucumis sativus* L.) seedlings. The effect of water deficiency in the RZ temperature treatments was also estimated by adding polyethylene glycol (PEG), which is often used to simulate drought stress in hydroponic systems [24,25]. In this study, cucumber was used as a model species because cucumber is a major commercial crop that is sensitive to both low temperature and water stress [26,27].

## Materials and Methods

### Plant material and growth conditions

**Fig 1:**
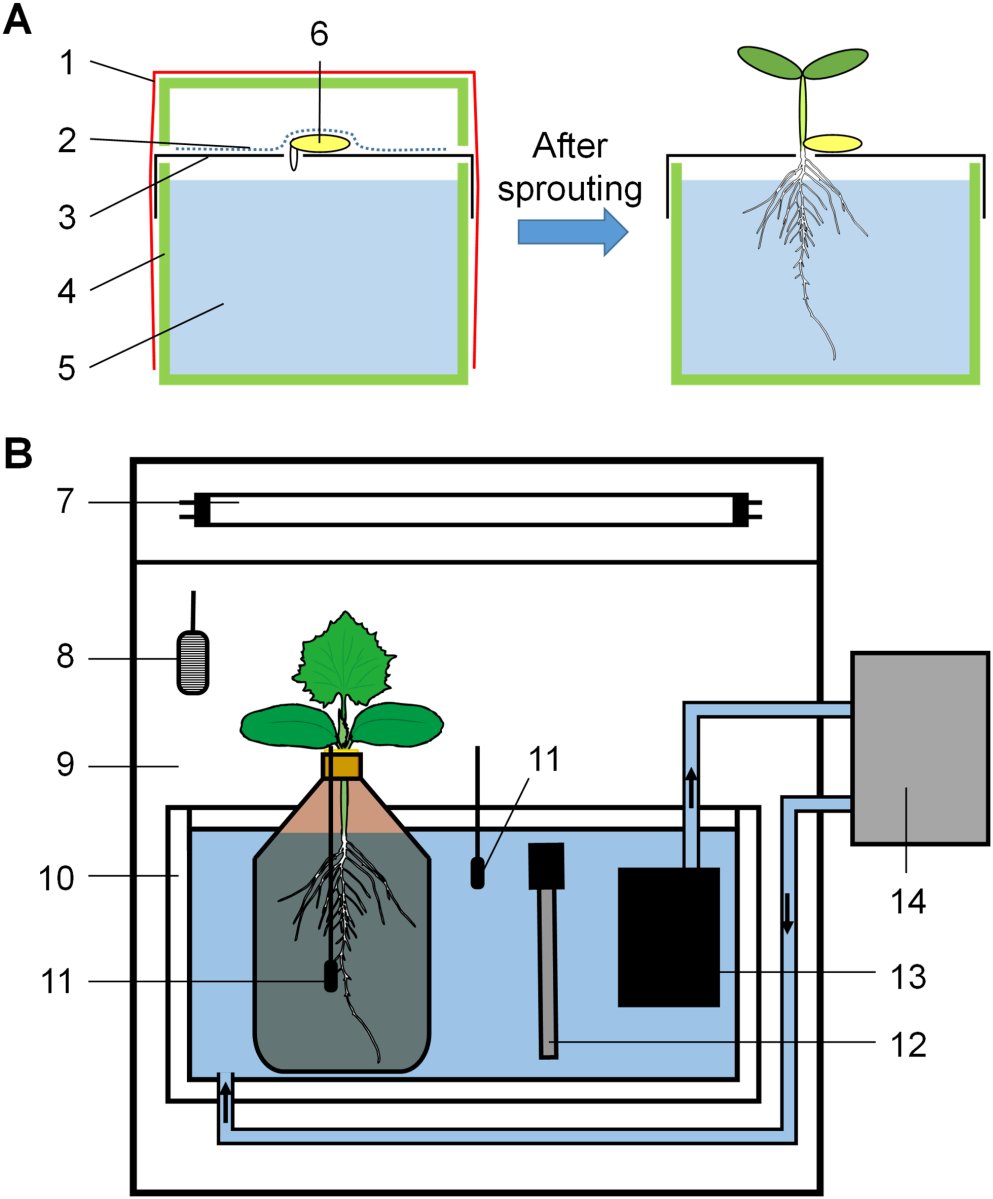
The scheme of cultivation devices used in this experiment. (A) The hydroponic seedling raising device: 1. plastic wrap; 2. moist paper towel; 3. perforated filter paper; 4. plastic box and its lid; 5. nutrient solution; 6. pregerminated cucumber seed; (B) The regulation system for air and root-zone (RZ) temperature: 7. fluorescent lamps; 8. air temperature thermocouple; 9. growth chamber; 10. polystyrene foam box; 11. water temperature thermocouple; 12. electrothermal rod; 13. circulating pump; 14. semiconductor refrigerator.

Cucumber (*Cucumis sativus* L. cv. Zhongnong No.16) seeds were pregerminated at 28°C for 26 hours, then sown onto hydroponic seedling-raising devices (Fig 1A) containing full-strength Yamazaki nutrient solution (pH was adjusted to approximately 6.0) [28] and cultured at 28°C for 30 hours. Germinated seedlings were then cultivated at day/night (10/14 h) temperatures of 26 /18°C and a relative humidity of 60–80% under photosynthetically active radiation (PAR) of approximately 100 μmol·m^-2^·s^-1^. On day 10 after germination, the seedlings were transplanted, with their hypocotyl and root in a 500-mL brown glass bottle (one seedling per bottle) containing nutrient solution. Bottles were placed in artificial light incubators (capacity: 430 L; RXZ-430E, Ningbo Jiangnan Instrument Factory, China) providing a daytime PAR of 250 μmol·m^-2^·s^-1^ (fluorescent lamps, 6400K white) for five days. The RZ warming treatment began on day 16 after germination when each seedling had both a fully developed true leaf and a newly unfolded young leaf.

### Root-zone treatments

On day 16 after germination, the bottles with seedlings were transferred to specially designed devices (Fig 1B) that could maintain different temperatures around both the shoots and roots of the seedlings. Each water bath tank was equipped with an electrothermal rod and connected to a semiconductor refrigerator (XH-LF162, XH-Electron, China) so that the solution temperature could be independently controlled. Air temperature was regulated by the light incubator itself.

There were three different temperature treatments. For the sub-optimal treatments, the air temperature was set at 20/12°C (day/night), and the water bath temperature (equivalent to the RZ temperature) was set at 13±1°C (abbreviated as S13) or 19±1°C (abbreviated as S19). For the optimal temperature treatments, the air temperature was set at 26/18°C (day/night), and the water bath temperature was set at 19±1°C (abbreviated as O19). Moreover, for each temperature treatment, half of the seedlings were also treated with 2% PEG dissolved in nutrient solution (S13+PEG, S19+PEG and O19+PEG). All treatments were continued until the second true leaf fully unfolded and the third leaf was just about to unfold, corresponding to a treatment period of 10 days for S13 (with or without PEG) and S19 (with or without PEG), and 5 days for O19 (with or without PEG). Forty seedlings per treatment were cultivated. On the last day of treatment, leaf gas exchange, Chl fluorescence and P700^+^ absorbance were measured. After the measurements, leaves were sampled for biochemical analyses. The remaining seedlings were used for growth characteristics analysis.

Air and RZ temperatures for each treatment were monitored and recorded by thermo recorders (RS-12 and RT-12, ESPEC MIC CORP., Japan; S1 Fig).

### Bleeding rate

On day 4 after treatment, four seedlings per treatment were cut below the cotyledons and the incision was quickly covered with a tubelet filled with absorbent cotton to collect bleeding sap for 3 hours. The bleeding rate was calculated from the weight increment of the tubelets after this period [19,29].

### Growth characteristics

Seedlings were harvested at both the beginning and end of the treatments (four replicates, four seedlings each replicate). All leaves were scanned (EPSON EXPRESSION 4990, Japan) and leaf area was calculated with WinRHIZO software (LC4800-II LA2400; Sainte-Foy, Canada). Plant dry weight was recorded after drying in an oven at 85°C for 48 h. Growth parameters were calculated as follows [30]:

$$\text{RGR}=\frac{\text{ln}W_{2}-\text{ln}W_{1}}{T_{2}-T_{1}}$$ (1)

$$\text{ULR}=\frac{W_{2}-W_{1}}{T_{2}-T_{1}}\cdot \frac{\text{ln}L_{A2}-\text{ln}L_{A1}}{L_{A2}-L_{A1}}$$ (2)

$$\text{LAR}=\frac{L_{A2}/W_{2}+L_{A1}/W_{1}}{2}$$ (3)

$$\text{AGR}=\frac{W_{2}-W_{1}}{T_{2}-T_{1}}$$ (4)

$$\text{SLA}=\frac{L_{A}}{W_{L}}$$ (5)

RGR is the mean relative growth rate over the treatment interval. ULR is the mean unit leaf ratio (also called the “net assimilation rate”, NAR). LAR is the mean leaf area ratio. AGR is the average growth rate over a treatment interval. SLA is the specific leaf area. *W*_1_ and *L*_*A*1_ are the dry weight and leaf area at time *T*_1_ (before treatment), *W*_2_ and *L*_*A*2_ are the dry weight and leaf area at time *T*_2_ (after treatment), and *W*_*L*_ is the dry weight of the same leaf.

### Leaf gas-exchange parameters and chlorophyll content

Gas-exchange was measured on two true leaves (four cucumber seedlings per treatment) with an LI-6400xt gas exchange analyser (Li-Cor 6400xt, Lincoln, NE, USA). Determination of the net assimilation rate versus intercellular CO_2_ concentration (*A*-*C*_i_) curves started from the third hour of a light period. The temperature of the measurement chamber (the block temperature) was set at the air temperature of the corresponding treatment, and the PAR and air relative humidity were maintained at 1200 μmol·m^-2^·s^-1^ and 60%-70%, respectively. The reference CO_2_ concentration was reduced from 400 to 320, 250, 200,150, 100 and 60 μmol CO_2_·mol^-1^, then increased from 60 to 400, 550, 750, 950, 1200 and 1500 μmol CO_2_·mol^-1^. The *A*-*C*_i_ curves were transformed to *A*-*C*_c_ curves (*C*_c_: calculated CO_2_ partial pressure at the sites of carboxylation [31]). The *A*-*C*_c_ curves were used to calculate the maximum rate of carboxylation (*V*_cmax_) and the electron transport rate at saturating PPFD contributing to RuBP regeneration (*J*_sat_), and then *V*_cmax_ and *J*_sat_ were calibrated to 25°C using software provided by Sharkey et al. [31]. The *A*-*C*_i_ (*A*-*C*_c_) curves calibrated to 25°C were also used to calculate the photosynthetic rate limitations imposed by *g*_s_ (*L*_s_) at *C*_a_ = 400 μmol CO_2_·mol^-1^ as described by ref. [32,33].

Leaf area was quickly scanned before chlorophyll (Chl) determination. After scanning, Chl was extracted by 95% ethanol and spectrophotometrically determined as described by Sartory and Grobbelaar [34].

### Rubisco activity and its protein subunits abundance assay

Rubisco activity measurements and its protein subunits abundance assays were performed as previously described [35]. The leaves were harvested on the last day of the treatment, immediately frozen in liquid nitrogen, and stored at -80°C until further analysis.

The total soluble protein concentration was determined at 595 nm with bovine serum albumin as the standard according to the method of Bradford [36].

To determine the relative amounts of Rubisco large and small subunits, protein extracts were separated by 12% SDS-PAGE and transferred onto a PVDF membrane. For immunoblotting, both rabbit polyclonal anti-RbcL antibody (AS03 037, Agrisera, Vännäs, Sweden, 1:3000) and anti-RbcS antibody (AS07 259, Agrisera, Vännäs, Sweden, 1:3000) were used to detect proteins [37]. Second antibody (goat anti-rabbit, HRP-conjugated) was used at a dilution of 1:5000, and the signal was detected by SuperSignal West Pico Chemiluminescent Substrate (Thermo Fisher, US). For all treatments, the abundances of Rubisco large and small subunits were normalized to those in O19 leaves.

### Chl fluorescence and P700^+^ absorbance measurements

Chl fluorescence and P700^+^ absorbance were measured with a Dual-PAM-100 system (Walz, Germany) [38] at the diurnal growth air temperature on two true leaves. Four cucumber seedlings from each treatment were used. After a night of dark adaptation and one hour of temperature adaptation, the minimum fluorescence (*F*_0_) was determined, then two saturating pulses were applied to obtain the maximum fluorescence (*F*_m_) and maximum P700^+^ signal (*P*_m_). After a 1-min dark period, actinic light (PAR = 221 μmol m^-2^ s^-1^) was applied for 8 min, and saturating light pulses were applied every 20 s to determine the maximum fluorescence signal (*F*^’^_m_) and maximum P700^+^ signal (*P*^’^_m_). A 1-s dark interval following each saturating pulse was used to determine the minimum level of P700^+^ signal (*P*_0_). The complementary quantum yields of PSII and PSI were calculated by Dual-PAM software [39–41] (Table 1). The electron transport rate (ETR) in the two photosystems was then calculated as indicated in Table 1.

**Table 1:** Glossary and formulas of Chl fluorescence, P700^+^ absorbance and the JIP-test parameters.

Biophysical parameters derived from Chl fluorescence and P700^+^ absorbance parameters	
$\Phi_{\text{II}}=(F_{\text{m}}^{'}-F)/F_{\text{m}}^{'}$	Quantum yield of photochemical energy conversion in PSII
_$\Phi_{\text{NPQ}}=F/F_{\text{m}}^{'}-F/F_{\text{m}}$_	Quantum yield of regulated non-photochemical energy loss in PS II
Φ_NO_ = *F* / *F*_m_	Quantum yield of non-regulated non-photochemical energy loss in PS II
$\Phi_{\text{I}}=(P_{\text{m}}^{\text{'}}-P)/P_{\text{m}}$	Quantum yield of photochemical energy conversion in PSI
Φ_ND_ = (*P* − *P*_0_) / *P*_m_	Quantum yield of non-photochemical quantum energy loss in PS I due to donor side limitation
$\Phi_{\text{NA}}=(P_{\text{m}}-P_{\text{m}}^{'})/P_{\text{m}}$	Quantum yield of non-photochemical quantum energy loss in PS I due to acceptor side limitation
ETR_II_ = Φ_II_×PAR×0.84×0.5	Electron transport rate in PSII
ETR_I_ = Φ_I_×PAR×0.84×0.5	Electron transport rate in PSI
Biophysical parameters derived from transient Chl fluorescence parameters	
*M*_0_ ≡ 4(*F*_300μs_ − *F*_50μs_) / (*F*_m_ − *F*_50μs_)	Approximated initial slope (in ms^−1^) of the fluorescence transient normalized on the maximal variable fluorescence F_V_
TR_0_/ABS ≡ *φ*_Po_ = 1−(*F*_0_ / *F*_m_)	Maximum quantum yield for primary photochemistry
ET_0_/TR_0_ ≡ *Ψ*_Eo_ = 1−*V*_J_	Efficiency/probability that an electron moves further than Q_A_^−^
ET_0_/ABS ≡ *φ*_Eo_ = [1−(*F*_0_ − *F*_m_)](1 − *V*_J_)	Quantum yield for electron transport
RE_0_/ET_0_ ≡ *δ*_Ro_ = (1 − *V*_I_) / (1 − *V*_J_)	Efficiency/probability with which an electron from the intersystem electron carriers is transferred to reduce end electron acceptors at the PSI acceptor side
RE_0_/ABS ≡ *φ*_Ro_ = [1−(*F*_0_ / *F*_m_)](1 − *V*_J_)	Quantum yield for reduction of end electron acceptors at the PSI acceptor side
RC/ABS = *φ*_Po_(*V*_J_ / *M*_0_)	Q_A_ reducing RCs per PSII antenna Chl
${\text{PI}}_{\text{ABS}}=\text{RC/ABS}\cdot \frac{\varphi_{\text{Po}}}{1-\varphi_{\text{Po}}}\cdot \frac{\psi_{\text{Eo}}}{1-\psi_{\text{Eo}}}$	Performance index (potential) for energy conservation from photons absorbed by PSII to the reduction of intersystem electron acceptors
${\text{PI}}_{\text{total}}{=\text{PI}}_{\text{ABS}}\cdot \frac{\delta_{\text{Ro}}}{1-\delta_{\text{Ro}}}$	Performance index (potential) for energy conservation from photons absorbed by PSII to the reduction of PSI end acceptors

Subscripts J and I denote J-step (2 ms) and I-step (30 ms) of OJIP, respectively.

The light induction transient of the Chl fluorescence (OJIP curve) was simultaneously recorded. Data in OJIP curves were converted to relative values as follows:

$$V_{\text{t}}=\frac{F_{\text{t}}-F_{0}}{F_{\text{m}}-F_{0}}$$ (6)

Where *V*_t_ and *F*_t_ represent the relative variable fluorescence and the fluorescence intensity at time t, respectively, and *F*_o_ and *F*_m_ denote the initial and the maximal fluorescence intensity, respectively. Further analysis of the OJIP curves, the so-called JIP-test, was conducted according to Strasser et al. [42–44]. Related glossary and formulas are provided in Table 1. All parameters were normalized using the corresponding values of O19 as reference, and then displayed in a radar map to more clearly depict the efficiencies for the whole energy cascade and the performance indexes [42].

### Statistical methods

All data shown represent the mean of at least three repetitions. The data and the graphs were processed using Microsoft Excel 2013. For multiple comparisons, data were subjected to one-way analysis of variance (ANOVA) and the means were compared using Tukey HSD tests at *P* = 0.05 (software: IBM SPSS Statistics 19.0, IBM Corporation, NY, USA). Two-way ANOVA was performed to compare sources of variation, including RZ temperature (RT), polyethylene glycol (PEG), and the RT×PEG interaction.

## Results

### Bleeding rates under different temperature conditions

**Fig 2:**
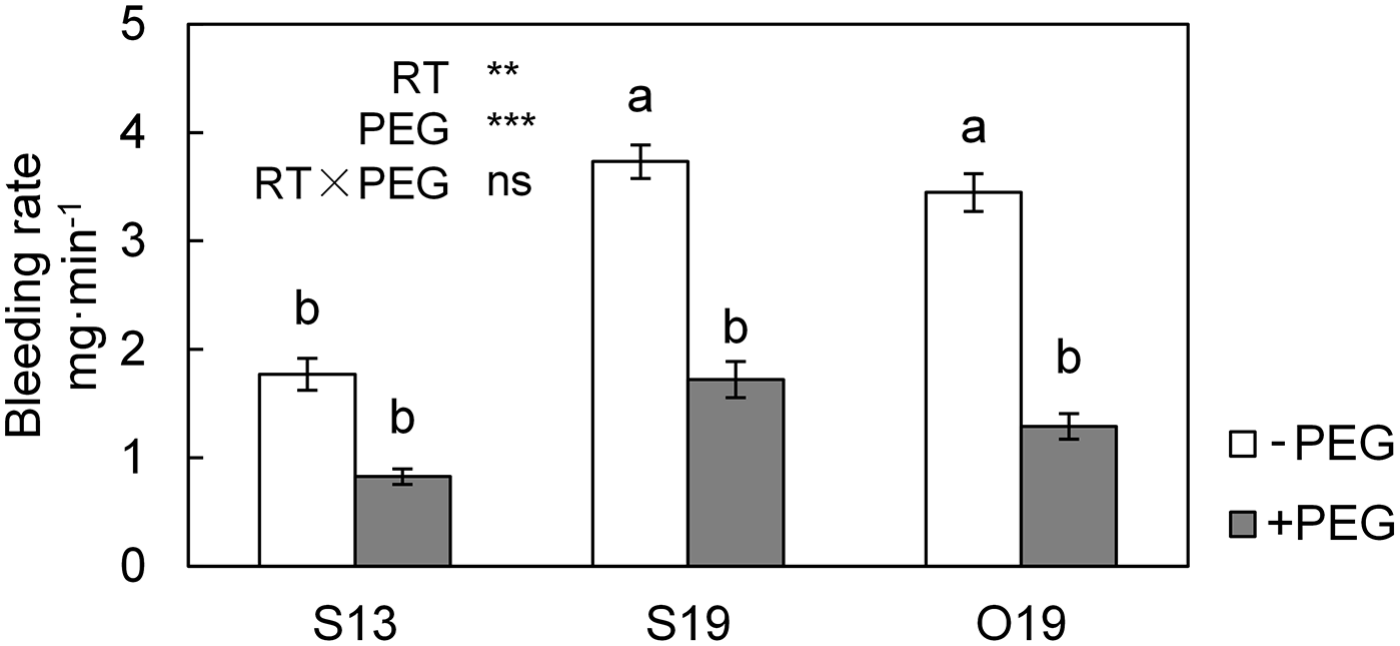
The bleeding rate of seedlings under different root-zone (RZ) conditions. All data are presented as means ± standard error (n = 3). Means with different letters denote significant difference (*P* < 0.05) by Tukey HSD. Source of variation: root-zone temperature (RT), polyethylene glycol (PEG), and RT×PEG interaction. ** *P* < 0.01; *** *P* < 0.001; ns: not significant.

As shown in Fig 2, when compared with O19, S13 significantly decreased the bleeding rate of cucumber seedlings by a half, whereas S19 did not exhibit any influence. PEG addition limited the bleeding rate in O19 and S19 to a similar extent as S13. Thus, a decrease of 6°C in RZ temperature (S13 versus S19 and O19) and 2% PEG addition exhibited similar effects on limiting the bleeding rate in this experiment.

### Plant growth parameters under different temperature conditions

**Fig 3:**
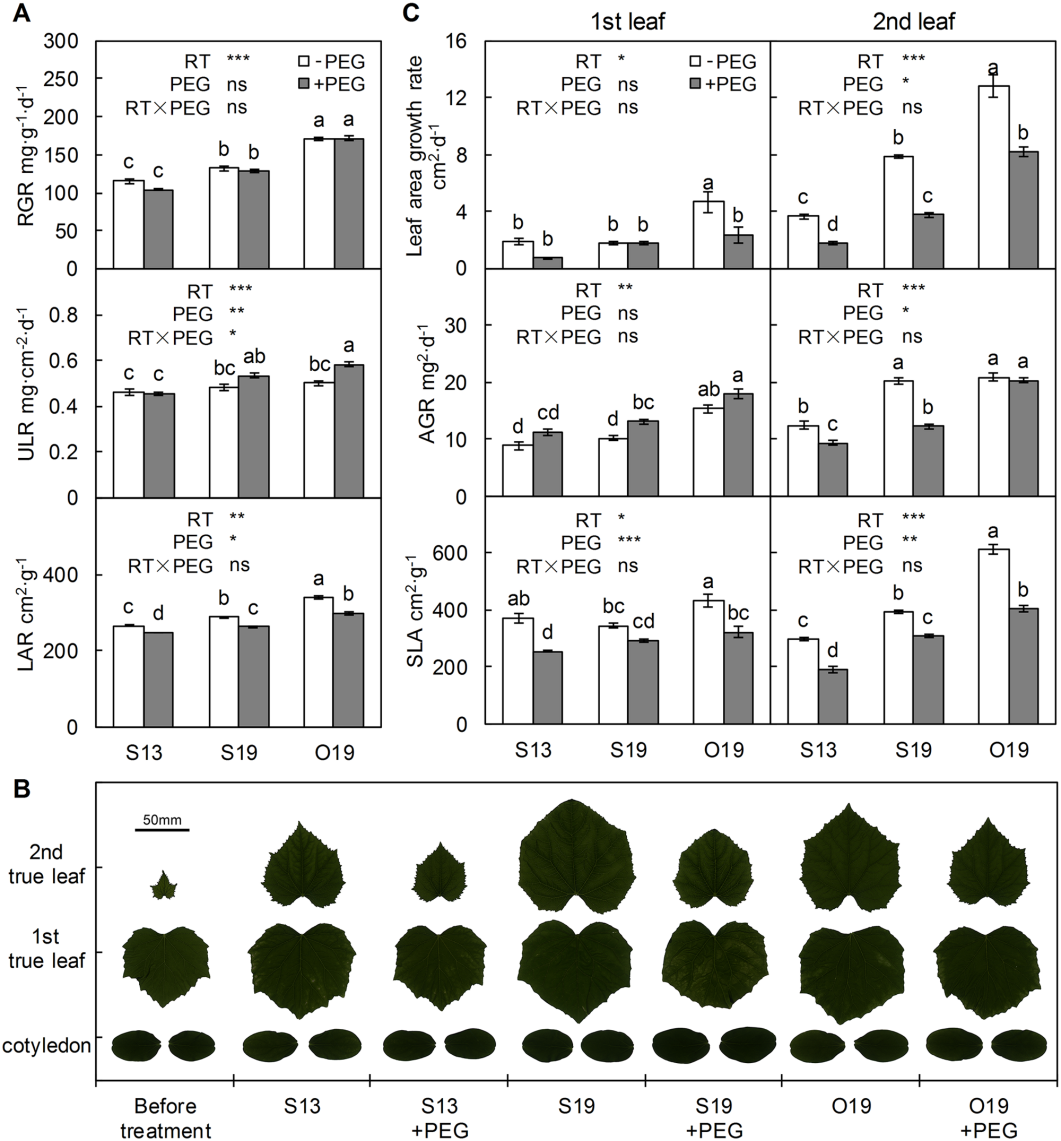
Plant growth characteristics under different root-zone (RZ) temperature and PEG treatments. (A) Relative growth rate (RGR) of the total dry mass, unit leaf rate (ULR) and leaf area ratio (LAR). (B) Scanned images of leaves. (C) Leaf area growth rate, dry mass average growth rate (AGR) and specific leaf area (SLA) of the 1st leaf and 2nd leaf. Means ± standard error are presented (n = 4). Means with different letters are significantly different (*P* < 0.05) by Tukey HSD. Source of variation: RZ temperature, RT; polyethylene glycol, PEG; and RT×PEG interaction; * *P* < 0.05; ** *P* < 0.01; *** *P* < 0.001; ns: not significant.

Compared with O19, the overall suboptimal temperature significantly decreased plant RGR (S13 with or without PEG addition, Fig 3A). Compared with S13, RZ warming (S19) significantly increased RGR by approximately 18%. However, PEG did not influence RGR (*P* > 0.05).

The RGR can be divided into two parts: ULR and LAR (Fig 3A). ULR was not influenced by temperature treatments in the absence of PEG but significantly increased in O19 and S19 treatments compared with S13 in the presence of PEG. LAR was significantly increased by S19 compared with S13, and by O19 compared with S19 in the absence of PEG. However, for all temperature treatments, PEG addition significantly decreased LAR, suggesting that the morphological characteristics of leaves are influenced by either RZ temperature or PEG addition.

In this experiment, two true leaves of each seedling were observed separately: the first true leaf, which was nearly mature before treatment, and the second true leaf, which was about to expand when the treatment started. These two leaves responded differently to different temperatures. The difference in leaf area induced by treatments was greater in the second leaf than in the first leaf (Fig 3B). Leaf area growth rates are shown in Fig 3C. The two true leaves grew more slowly under sub-optimal temperature conditions. For the first leaf, area growth was generally less evident during treatment compared to the newly unfolded leaf. The leaf area growth rate of seedlings in O19 was significantly higher than that in any other treatment. The area growth rate of the second leaf was significantly improved by increasing the RZ temperature; the increase was nearly two fold in S19 and more than threefold in O19 treatment compared with S13. However, the addition of PEG counteracted this effect in S19 and O19, as shown in Fig 3C, and a significant decrease in the leaf area growth rate was observed in S13 in the presence of PEG. With respect to the dry mass increment, compared with S13, S19 did not affect AGR of the first leaf, but significantly increased AGR of the second leaf to the level of O19 (Fig 3C). PEG treatments increased the mean AGR of the first leaf in each temperature treatment (significantly in S19), but significantly decreased the AGR of the second leaf in the seedlings exposed to both S13 and S19 treatments. Because PEG had no significant influence on RGR (Fig 3A), restricted expansion of the second leaf may lead to allocation of new dry mass mainly to other organs, such as the first leaf. RZ warming did not influence SLA of the first leaf but significantly increased SLA of the second leaf in S19 compared with S13 (Fig 3C). In all treatments, PEG addition significantly decreased SLA of the true leaves, except for the first leaf of S19 seedlings. The observed variation of SLA can be used to compare the effect of all treatments on leaf area with leaf weight. Thus, the above results indicate that in the second leaf, the positive effect of RZ warming and the negative effect of PEG addition were stronger for leaf area than for leaf weight.

### Photosynthetic parameters under different temperature conditions

In the first true leaf, the net CO_2_ assimilation rate (*A*_400_) was low when seedlings were maintained in an overall sub-optimal temperature environment (S13, Table 2). However, *A*_400_ was significantly increased by RZ warming (S19 versus S13) and reached the levels measured under optimal conditions (O19). *V*_cmax_ and *J*_sat_, which partially represent non-stomatal limitation of leaf photosynthesis, exhibited similar trends as *A*_400_, indicating that both the dark and light reactions of photosynthesis were promoted by RZ warming treatment (S19 versus S13). The extent of stomatal limitation was also evaluated (*g*_s_, *C*_i_ and *L*_s_). Severe stomatal closure was observed in overall sub-optimal temperature-treated seedlings (S13), leading to high *L*_s_ and a low Tr. This negative condition was partially alleviated by RZ warming (S13 versus S19 and O19). The lack of a significant difference in *C*_i_ between S19 and S13 seedlings indicates that non-stomatal and stomatal limitation were alleviated to a similar extent by S19. PEG treatments exhibited a smaller influence on photosynthesis than RZ temperature, and almost no effect on stomatal conductance or transpiration rate (except O19), although there was a non-significant trend in which PEG addition increased *C*_i_ and decreased *L*_s_, indicating that PEG addition might result in greater non-stomatal limitation than stomatal limitation.

**Table 2:** Gas-exchange parameters in the two true leaves of cucumber seedlings under different root-zone (RZ) temperature and PEG treatments.

Treatment	*A*_400_ μmol CO_2_·m^-2^·s^-1^		*V*_cmax_ μmol·m^-2^·s^-1^		*J*_sat_ μmol·m^-2^·s^-1^		*g*_s_ mol H_2_O·m^-2^·s^-1^		*C*_i_μmol·mol^-1^		*L*_s_		Trmmol H_2_O·m^-2^·s^-1^	
**1st leaf**														
S13	6.2	c	45	b	99	d	0.05	b	281	c	0.38	a	0.76	c
S13+PEG	4.7	c	41	b	103	cd	0.06	b	320	ab	0.32	ab	0.41	c
S19	17.6	a	78	a	141	a	0.13	ab	267	c	0.27	bc	1.50	b
S19+PEG	12.9	b	52	b	121	b	0.13	ab	297	bc	0.23	c	1.52	b
O19	18.4	a	71	a	119	bc	0.25	a	316	ab	0.09	d	2.88	a
O19+PEG	16.5	a	67	a	122	b	0.26	a	336	a	0.13	d	1.96	b
**Source of variation**														
RT	0.000		0.000		0.000		0.003		0.493		0.000		0.000	
PEG	0.007		0.010		0.873		0.918		0.032		0.607		0.134	
RT×PEG	0.288		0.151		0.387		0.831		0.387		0.432		0.654	
Adjusted R^2^	0.927		0.844		0.589		0.338		0.145		0.546		0.636	
**2nd leaf**														
S13	9.6	c	50	bc	107	a	0.07	cd	270	b	0.33	ab	0.83	b
S13+PEG	4.0	d	35	d	83	c	0.03	d	277	b	0.43	a	0.42	b
S19	13.0	b	45	cd	85	bc	0.19	b	328	a	0.14	cd	1.88	a
S19+PEG	12.3	b	48	cd	101	abc	0.13	c	289	b	0.23	bc	1.01	b
O19	16.9	a	66	a	104	ab	0.28	a	339	a	0.05	d	2.39	a
O19+PEG	16.9	a	60	ab	110	a	0.26	ab	336	a	0.11	d	2.41	a
**Source of variation**														
RT	0.000		0.015		0.045		0.000		0.000		0.000		0.000	
PEG	0.007		0.759		0.660		0.161		0.528		0.010		0.126	
RT×PEG	0.013		0.328		0.021		0.946		0.166		0.647		0.972	
Adjusted R^2^	0.799		0.233		0.318		0.621		0.640		0.792		0.572	

Net CO_2_ assimilation rate at *C*_a_ = 400 μmol·mol^-1^ (*A*_400_); maximum rate of carboxylation (*V*_cmax_); electron transport rate at saturating PPFD (*J*_sat_); stomatal conductance (*g*_s_); intercellular CO_2_ concentrations (*C*_i_) at *C*_a_ = 400 μmol·mol^-1^; relative limitation posed by stomatal conductance (*L*_s_) and transpiration rate (Tr). All data except *g*_s_ and Tr, were calibrated to 25°C estimated from *A*/*C*_c_ curves. Means with different letters are significantly different (*P* < 0.05, n = 3 or 4) by Tukey HSD. Source of variation: *P* values of root-zone temperature (RT), polyethylene glycol (PEG) and RT×PEG interaction.

In the second true leaf, S19 had no positive effect on *V*_cmax_ and *J*_sat_ compared with S13 but promoted *g*_s_ and *A*_400_. The increase in *C*_i_ (S19 versus S13) induced by RZ warming indicates the alleviation of stomatal limitation in the second leaf. PEG treatment differently influenced photosynthesis in the second leaf in S13, S19 and O19 seedlings. In S13+PEG seedlings, the development of the second leaf was extremely suppressed, as shown in Fig 3B, and its *A*_400_ decreased mainly due to the low *V*_cmax_ and *J*_sat_. In S19+ PEG seedlings, the decrease in *A*_400_ was more due to stomatal limitation (*g*_s_ and *L*_s_, Table 2), similar to S13 seedlings; in O19+PEG seedlings, *A*_400_ was not affected. The transpiration rates of the second leaf showed similar trends with *g*_s_ in all the treatments.

Data on Rubisco activity and amount are presented in Table 3. In the first true leaf, the initial and total Rubisco activity exhibited similar trends, these parameters increased in the order S13<S19<O19. The Rubisco activation rate did not differ significantly among the different treatments. The translation of both the large and small subunits of Rubisco was reduced at sub-optimal air temperature; however, RZ warming did not significantly change these parameters. In the second true leaf, neither the activity nor amount of Rubisco was influenced by RZ warming under sub-optimal temperature conditions. The relative values of the initial and total Rubisco activities in the sub-optimal temperature treatments were higher in the second true leaf than in the first true leaf, indicating acclimation in the second true leaf. Similarly, when comparing S13 seedlings with S19 and O19 seedlings, the total chlorophyll was significantly lower in the mature leaf but unchanged in the newly unfolded leaf. Likewise, the soluble protein was not different in the mature leaf but significantly higher in the newly unfolded leaf. PEG treatment usually resulted in no effect or a slightly negative effect on the biochemical parameters in both leaves, except in the second leaf of S13+PEG seedlings, in which Chl and protein concentration were significantly reduced.

**Table 3:** Rubisco properties, total chlorophyll and soluble protein in cucumber seedling leaves under different root-zone (RZ) temperature and PEG treatments.

Treatment	Rubisco initial activity μmol·g^-1^ protein·^-1^min*		Rubisco total activity μmol·g^-1^ protein·^-1^min		Rubisco activation rate %		Rubisco large subunits relative abundance		Rubisco small subunits relative abundance		Total Chl μg·cm^-2^		Total soluble protein mg·g^-1^FW	
**1st leaf**														
S13	208(63)	c	251(67)	c	83.0%	a	1.39	a	1.56	a	36.7	bc	18.9	a
S13+PEG	199(60)	c	243(64)	c	81.6%	a	1.37	a	1.38	a	32.6	c	17.9	a
S19	275(84)	ab	327(87)	b	84.0%	a	1.58	a	1.62	a	46.6	a	18.8	a
S19+PEG	255(78)	bc	300(80)	b	84.9%	a	1.45	a	1.40	a	47.3	a	20.8	a
O19	329(100)	a	377(100)	a	87.0%	a	1.85	a	1.91	a	51.6	a	19.7	a
O19+PEG	296(90)	ab	340(90)	ab	87.0%	a	1.52	a	1.47	a	43.3	ab	20.8	a
**Source of variation**														
RT	0.000		0.000		0.110		0.102		0.255		0.000		0.039	
PEG	0.131		0.195		0.695		0.348		0.095		0.106		0.474	
RT×PEG	0.229		0.347		0.871		0.473		0.903		0.616		0.464	
Adjusted R^2^	0.742		0.668		0.004		0.125		0.120		0.585		0.167	
**2nd leaf**														
S13	225(83)	bc	310(93)	ab	72.6%	ab	1.83	a	1.67	a	45.0	a	24.6	a
S13+PEG	191(71)	c	268(80)	b	71.1%	b	1.57	a	1.50	a	20.0	c	15.2	c
S19	236(87)	ab	318(95)	a	74.0%	ab	1.61	a	1.79	a	42.2	ab	19.7	b
S19+PEG	224(83)	bc	314(94)	a	71.4%	ab	1.80	a	1.83	a	38.5	ab	20.7	b
O19	270(100)	a	334(100)	a	81.0%	a	1.84	a	1.78	a	41.9	ab	20.4	b
O19+PEG	273(101)	a	346(104)	a	79.2%	ab	1.65	a	1.72	a	34.6	b	21.0	b
**Source of variation**														
RT	0.005		0.003		0.041		0.590		0.124		0.000		0.350	
PEG	0.051		0.125		0.599		0.634		0.239		0.000		0.000	
RT×PEG	0.033		0.126		0.802		0.630		0.819		0.000		0.000	
Adjusted R^2^	0.453		0.421		0.116		-0.150		0.069		0.878		0.747	

Means with different letters are significantly different (*P* < 0.05, n = 3 or 4) by Tukey HSD. Source of variation: *P* values of root-zone temperature (RT), polyethylene glycol (PEG) and RT×PEG interaction.

*: the numbers in parentheses indicate the percentages of the control (O19).

Due to the apparent acclimation of photosynthetic non-stomatal limitation in the second true leaf of S13 seedlings, in the following results, the reported data are solely from the first true leaf unless otherwise stated. The data for the second true leaf are provided in the supporting information (S1 and S2 Tables).

### Chl fluorescence and P700^+^ level under different temperature conditions

**Fig 4:**
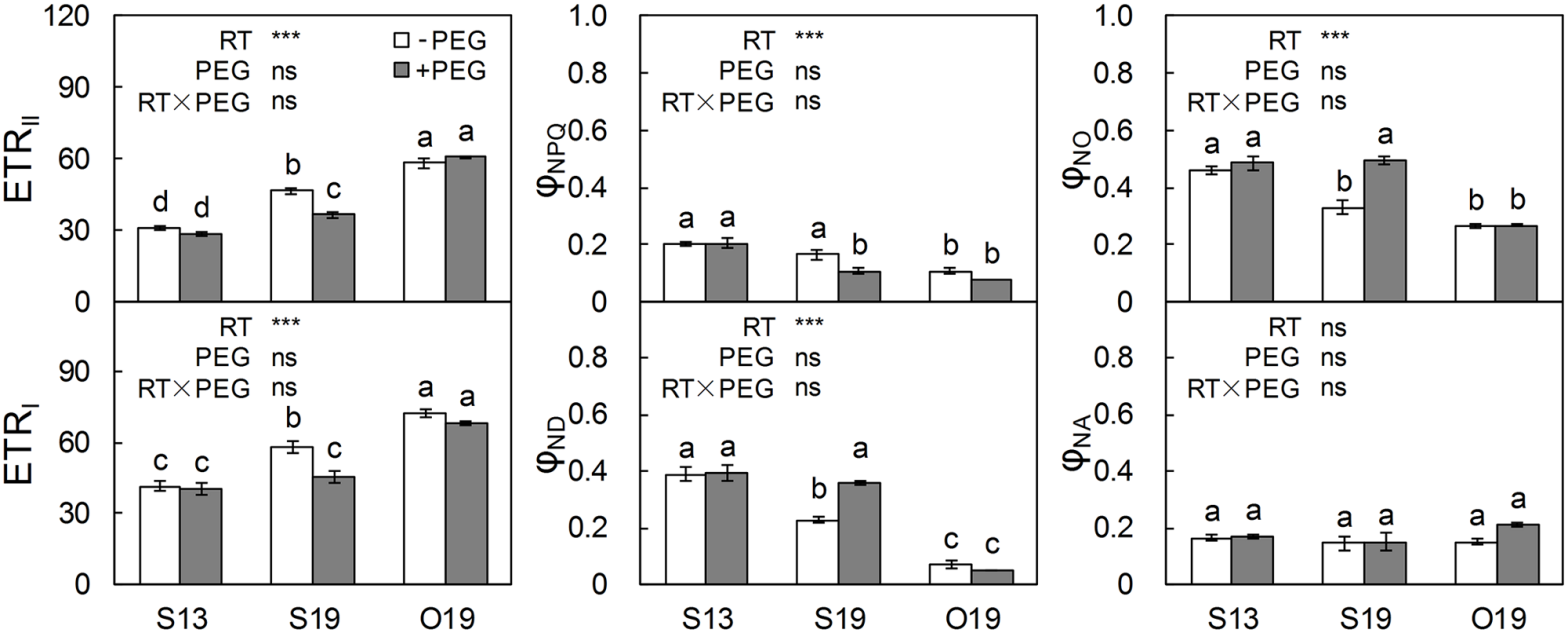
Chl fluorescence and P700^+^ parameters in the first true leaves under different root-zone (RZ) temperature and PEG treatments. Electron transport rate of PSII (ETR_II_), quantum yield of light-induced non-photochemical fluorescence quenching (Φ_NPQ_) of PSII and quantum yield of non-light-induced non-photochemical fluorescence quenching (Φ_NO_); electron transport rates of PSI (ETR_I_), quantum yield of non-photochemical energy dissipation in PSI due to donor-side limitations (Φ_ND_) and quantum yield of non-photochemical energy dissipation in PSI due to acceptor-side limitations (Φ_NA_). Means ± standard error are presented (n = 3 or 4). Means with different letters are significantly different (*P* < 0.05) by Tukey HSD. Source of variation: root-zone temperature, RT; polyethylene glycol, PEG; and RT×PEG interaction; *** *P* < 0.001; ns: not significant.

Sub-optimal temperature induced a low electron transport rate of the two photosystems in the first true leaf (S13 versus O19, Fig 4). S19 significantly increased ETR_II_ and ETR_I_ by 50% and 40%, respectively, compared with S13. ETR_I_ / ETR_II_ was 1.34, 1.25, and 1.24 in S13, S19 and O19 seedlings, respectively, and did not differ significantly. In PSII, Φ_NPQ_ did not change in S19 compared with S13, whereas Φ_NO_ significantly decreased in S19 to the level in O19, indicating less excess energy. As for PSI, Φ_ND_ in S19 seedlings was only 58% of that in S13, whereas Φ_NA_ exhibited no obvious difference among all treatments, indicating that PSI was limited mainly at the donor side. PEG treatment reduced most of the fluorescence parameters of S19 seedlings to the level of S13 but had no effect on either S13 or O19 seedlings.

### Light induction of Chl fluorescence transient and JIP-test parameters under different temperature conditions

**Fig 5:**
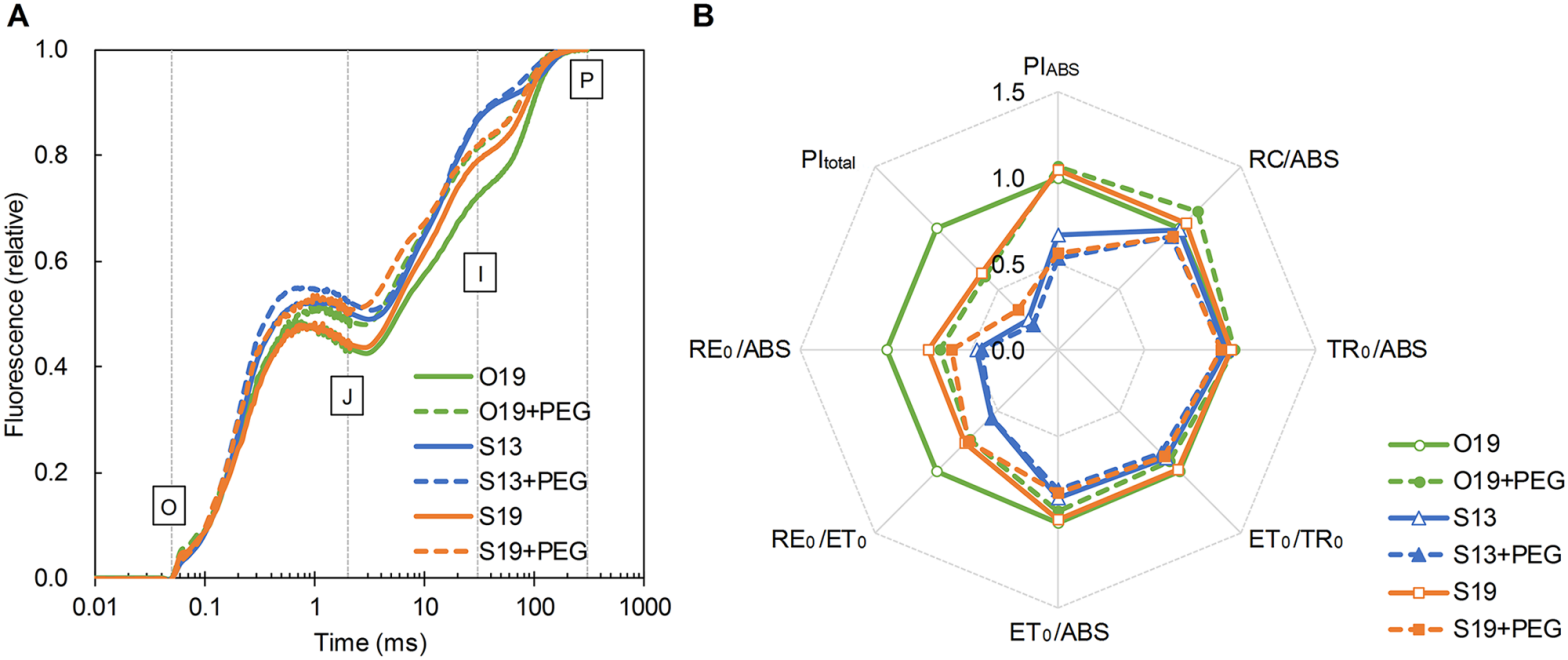
Light induction curves of transient Chl fluorescence (A) and JIP-test results (B). Data were determined in the first true leaves of cucumber seedlings under different root-zone (RZ) temperature and PEG treatments. RC/ABS: Q_A_ reducing RCs per PSII antenna Chl; TR_o_/ABS: maximum quantum yield for primary photochemistry; ET_o_/TR_o_: efficiency/probability that an electron moves further than Q_A_^−^; ET_o_/ABS: quantum yield for electron transport; RE_o_/ET_o_: efficiency/probability with which an electron from the intersystem electron carriers is transferred to reduce end electron acceptors at the PSI acceptor side; RE_o_/ABS: quantum yield for reduction of end electron acceptors at the PSI acceptor side; PI_ABS_: performance index (potential) for energy conservation from photons absorbed by PSII to the reduction of intersystem electron acceptors; PI_total_: performance index (potential) for energy conservation from photons absorbed by PSII to the reduction of PSI end acceptors. Lines and values are the averages of four individual measurements with four different seedlings.

Chl fluorescence induction curves (OJIP curves, Fig 5A) were recorded to investigate the location at which electron transport was blocked between PSII and PSI. The high J-level and I-level in S13 seedlings were decreased by RZ warming under sub-optimal temperature conditions (S19 versus S13), and the I-level was further decreased by O19. Adding PEG generally increased both the J-level and I-level in all temperature treatments. The JIP-test parameters were derived from quantitative analysis of the OJIP curves (Fig 5B). RC/ABS and TR_0_/ABS were not significantly different among the temperature treatments. Compared with S13, S19 significantly increased the efficiency parameters (ET_0_/TR_0_ and RE_0_/ET_0_) and, consequently, quantum yields (ET_0_/ABS and RE_0_/ABS). These increases in S19 resulted in a PI_ABS_ similar to O19 and a PI_total_ of 64% relative to O19, indicating significantly improved overall performance compared with S13 (67% and 25%, respectively). PEG addition did not affect S13 seedlings but inhibited PI_ABS_ and PI_total_ by decreasing ET_0_/TR_0_ in S19 seedlings and inhibited PI_total_ by decreasing both ET_0_/TR_0_ and RE_0_/ET_0_ in O19 seedlings.

## Discussion

In general, sub-optimal temperature suppresses plant growth by influencing both morphological properties and physiological activity of the leaves [1,45,46]. In this experiment, an increment of 6°C in RZ warming treatment (S13 versus S19) had a significant positive effect on the growth of cucumber seedlings (RGR, Fig 3A). This not only preserved the photosynthetic capability of the already existing leaf but also promoted the expansion rate of the newly developed leaf (Table 2 and Fig 3C). However, the phenology of the seedlings was not influenced by RZ temperature. It required 10 days for the second true leaf to fully unfold in sub-optimal air temperature treatments, but only 5 days in the optimal air temperature treatments. The negligible contribution of RZ warming to new leaf emergence may be the main reason that the RGR was lower in S19 seedlings than in O19 seedlings (Fig 3A). However, RZ warming obviously promoted new leaf expansion (Fig 3B).

Root-zone temperature regulation always leads to simultaneous changes in the root-sourced water supply [10,16,19], but differentiating the roles of root-sourced water signals and temperature signals is difficult. We provided a rough solution to this problem in this experiment by adding PEG to decrease the bleeding rate of S19/O19 seedlings to a level similar to that in S13 seedlings (Fig 2). The failure of PEG addition to influence RGR indicates that the improved root water availability at warmer RZ temperature did not affect total biomass accumulation. By contrast, PEG addition decreased the LAR and the growth rate of the newly unfolded leaf in S19 seedlings to a level similar to that in S13 seedlings (Fig 3A and 3C). The slight artificial water stress also decreased new leaf expansion in S13 and O19+PEG seedlings. These results are consistent with the work of Poire et al. [10], who observed that leaf expansion of *Ricinus communis* was strongly inhibited at a low RZ temperature during the day; they hypothesized that the decreased water supply due to root-cooling played a leading role. Similar results were also observed in rice plants [47], the leaf area expansion and transpiration were simultaneously reduced by 13°C root temperature.

PEG addition also provided insights on the ULR results. ULR did not differ significantly between S19 and S13 seedlings, indicating equilibria of the suppression / promotion of both total leaf area and total biomass accumulation in RZ warming. However, in S19+PEG seedlings, where the effect of water supply was excluded via the addition of PEG to isolate the effect of temperature, the ULR was significantly higher than in S13 seedlings. As shown in Fig 3B and 3C, the leaf area was similar in S19+PEG and S13 seedlings, and thus the increment of ULR in S19+PEG seedlings was mainly due to increased total biomass accumulation. This result further indicates that a warm RZ temperature itself rather than the improved root-sourced water supply can increase the net assimilation rate. This inference is also supported by the negligible effects of PEG on RGRs.

A number of studies have demonstrated that unfavorable RZ temperature depresses photosynthesis under normal air temperature conditions [9,48–51]. In this experiment, for the newly unfolded leaf, the photosynthetic activity of S13 seedlings was suppressed mainly due to stomatal limitation (Table 2), rather than metabolic limitation (*V*_cmax_ and *J*_sat_ in Table 2, Rubisco-related parameters in Table 3, and S1 and S2 Tables). Thus RZ warming treatment exhibited no significant benefits for this aspect. Similar acclimation of photosynthesis has been observed in Arabidopsis leaves developing at 5°C and was attributed to increases of available phosphates and the activity of several Calvin-cycle and sucrose-synthesis related enzymes [52]. A low RZ temperature alone may have also contributed to acclimation in a study by Venzhik et al. [51] in which wheat leaves exhibited decreased leaf area but increased cold tolerance after exposure to 2°C root chilling. Thus we suggest that for newly unfolded leaves during sub-optimal temperature stress, RZ warming benefits photosynthetic ability mainly via stomata opening rather than metabolic processes such as Rubisco carboxylation activity and electron transport rate.

Photosynthetic activity was severely impaired in the first leaf after transfer to a sub-optimal environment from an optimal temperature environment. Increasing the RZ temperature by 6°C (S19) maintained *A*_400_ at the high level of O19 (Table 2) by alleviating both stomatal and non-stomatal limitation (S19 seedlings versus S13). Stomatal apertures can be finely modulated by various environmental conditions such as hormonal stimuli, light signals, water status, CO_2_, and air temperature [53,54]. Non-optimal RZ temperature is also considered a cause of stomatal closure [14,49,55]. In addition to water supply, signals from the root such as ABA (abscisic acid) have also been identified as playing an important role in studies of RZ temperature [23,56]. For instance, Zhang et al. [50] demonstrated that in six *Cucurbitaceae* species at ambient air temperature, the change in the net photosynthesis rate was mainly due to the change in *g*_s_, which was regulated by RZ temperature via root-sourced ABA. In our experiment, additional PEG treatment had a very limited effect on *g*_s_ or Tr of both leaves, even though it altered bleeding rates significantly. These results suggest a potentially more important role of root-sourced temperature signals than water signals on stomatal aperture under the conditions of our experiment. The effects of phytohormones in RZ temperature regulation should be investigated in related further studies.

The metabolic photosynthetic potential excluding stomatal limitation is reflected by *V*_cmax_ and *J*_sat_ estimated from the *A*-*C*_c_ curves [57]. The decline of *V*_cmax_ in S13 seedlings was consistent with the trend of Rubisco activity (Tables 2 and 3). Li et al. [58] reported decreases in both Rubisco content and activity in cucumber leaves under sub-optimal temperature conditions, which were ascribed to the H_2_O_2_- and glutathione-mediated cell redox state. Tezara et al. [59] demonstrated that under water stress, suppression of photosynthetic assimilation is mainly because the loss of ATP synthase (coupling factor) induces inhibition of ribulose bisphosphate synthesis, which determines *J*_sat_ [31]. Lack of ATP, or a decreased ATP/ADP ratio may also inactivate Rubisco-activase and then decrease Rubisco activity [60,61]. Notably, ATP synthase is preferentially attacked by singlet oxygen in a conditional mutant *flu* of *Arabidopsis* [62]. Thus, the source of the decline in metabolic potential could be the light reactions, where reactive oxygen species (ROS) will be generated once photoinhibition occurs.

At a sub-optimal air temperature 20/12°C, photosynthetic electron transport in the first leaf was greater in S19 than in S13 seedlings due to RZ warming (Fig 4). PSII function was improved by S19 (healthy TR_0_/ABS and improved ET_0_/TR_0_; Fig 5B). Additionally, Φ_NPQ_ was not induced by the low air-temperature in either S13 or S19 seedlings, resulting in a higher proportion of Φ_NO_ in S13 than in S19 seedlings. The relatively high Φ_NO_ may lead to excess energy which will stimulate the generation of ROS [63,64]. Venzhik et al. [51] interpreted the reduced rate of electron transport in leaves of root-chilled seedlings as a result of adaptive transformations of the chloroplast membrane complex. This interpretation is consistent with our conditions, in which RZ warming may help mitigate such passive defence. Another aspect is nutrient condition. A cool RZ temperature may disturb the absorption of minerals such as N, Mg, Mn, and Zn [6,15], which are the core elements of the photosynthetic apparatus and antioxidant enzymes. In a newly unfolded leaf, this nutrient deficiency can be partially alleviated by decreasing its growth rate and receiving nutrients transferred from older tissues. This is a potential interpretation for the adaption occurring in the newly unfolded leaf.

The occurrence of PSI photoinhibition under both chilling and light intensity stress is an important feature in *Cucumis* plants [27,65]. However, in this experiment, the daytime air temperature (20°C) was higher than the level (10°C) below which PSI photoinhibition is usually observed. Moreover, the phenomenon of greater induction of Φ_ND_ rather than Φ_NA_ is also different from typical PSI photoinhibition. The increase in Φ_ND_ in S13 seedlings together with reduced RE_0_/ET_0_, which refers to the efficiency of electron movement from the PQ pool to the PSI end acceptors [44], suggests that electron transport between PQH_2_ and cytochrome (cyt) b_6_/f might have been partially blocked or bypassed under overall sub-optimal temperature environment. This phenomenon might be related to stimulated plastid activity of terminal oxidase (PTOX), which has been suggested to act as a safety valve to prevent over-reduction of the electron transfer chain [66,67].

Although chilling or sub-optimal temperature-induced photoinhibition has been observed in various plant species [68–72], the interaction between RZ temperature and photosynthetic electron transport has rarely been further investigated. No consensus on RZ temperature effects has been achieved, possibly due to differences in evaluated species, treatment intensities or sampling location. For example, in wheat seedlings at an air temperature of 22°C, a 2°C RZ temperature decreased ETR_II_ and increased the coefficient of non-photochemical quenching (qN) [51]. In cucumber plants exposed to ambient air temperature in a study by Zhang et al. [50], a 14°C RZ temperature did not induce any photoinhibition. In rice seedlings at a chilling air temperature of 10°C, a 25°C warm root temperature severely blocked electron transport in leaves and led to visible damage [20]. In our preliminary experiment, a 23°C RZ temperature treatment performed poorly at 20/12°C sub-optimal air temperature and was abandoned. Although 20–25°C is considered an optimal temperature range for cucumber roots under ambient air temperature conditions [7,73], the definition of “optimal root temperature” should be adjusted once the shoot temperature is changed. The results described above indicate the complexity of shoots/roots interactions in plant responses to the surrounding temperature, and underline the necessity of specifying the air temperature when evaluating the effects of RZ temperature regulation.

## Conclusion

Our results demonstrate that moderate RZ warming can increase RGR of cucumber seedlings under sub-optimal air temperature but does not influence new leaf emergence, leading to a reduced RGR compared with overall optimal temperature conditions. The promotion of growth by RZ warming consists of two different aspects in mature and newly unfolded leaves. In mature leaves, RZ warming substantially increases the net photosynthesis rate to the level measured under the optimal temperature condition by alleviating both diffusive and metabolic limitation. In the newly unfolded leaves, RZ warming significantly promotes leaf area expansion and reduces the stomatal limitation of photosynthesis. RZ warming also improved the root-sourced water supply, with a greater effect on new leaf expansion than on photosynthesis.

## Supporting Information

S1 Fig Typical daily air and root-zone temperature fluctuations in different treatments. (TIF)

S1 Table Chl fluorescence and the P700^+^ parameters in the second true leaves under different root-zone temperature and PEG treatments. (DOCX)

S2 Table The JIP-test results in the second true leaf of cucumber seedlings under different root-zone temperature and PEG treatments. (DOCX)
