# Supplementary figures and images for: Root-Zone Warming Differently Benefits Mature and Newly Unfolded Leaves of Cucumis sativus L. Seedlings under Sub-Optimal Temperature Stress
Source: PLoS One. 2016 May 6;11(5):e0155298. doi: 10.1371/journal.pone.0155298 (PMC4859567; doi:10.1371/journal.pone.0155298)

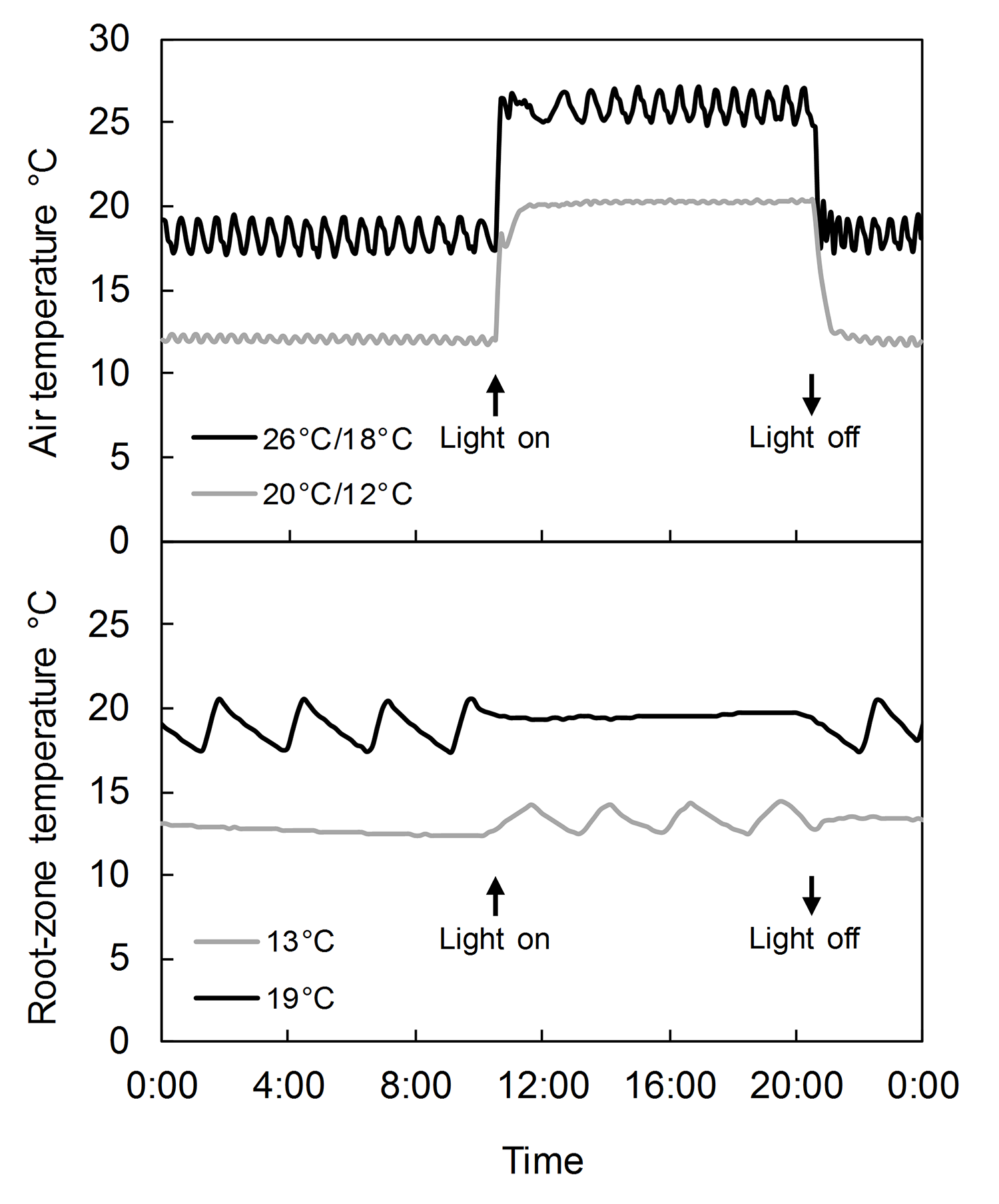

Supplement: S1 Fig — (TIF) [file pone.0155298.s001.tif]
