# Supplementary material for: Root-Zone Warming Differently Benefits Mature and Newly Unfolded Leaves of Cucumis sativus L. Seedlings under Sub-Optimal Temperature Stress
Source: PLoS One. 2016 May 6;11(5):e0155298. doi: 10.1371/journal.pone.0155298 (PMC4859567; doi:10.1371/journal.pone.0155298)
Supplement: S1 Table — (DOCX) [file pone.0155298.s002.docx]

**S1 Table.** **Chl fluorescence and the P700^+^ parameters in the second true leaves under different root-zone temperature and PEG treatments.** Means with different letters denote significant difference (*P* < 0.05, n = 3 or 4) by Tukey HSD.

| Treatment | ETR_II_ | | ETR_I_ | | ETR_I_ / ETR_II_ | | Φ_NPQ_ | | Φ_NO_ | | Φ_ND_ | | Φ_NA_ | |
| --- | --- | --- | --- | --- | --- | --- | --- | --- | --- | --- | --- | --- | --- | --- |
| S13 | 46.9 | bc | 53.3 | c | 1.22 | a | 0.140 | ab | 0.355 | bc | 0.284 | a | 0.141 | b |
| S13+PEG | 38.0 | d | 42.3 | d | 1.30 | a | 0.124 | bc | 0.467 | a | 0.297 | a | 0.247 | a |
| S19 | 41.3 | cd | 49.7 | cd | 1.21 | a | 0.177 | a | 0.377 | b | 0.338 | a | 0.126 | b |
| S19+PEG | 42.3 | cd | 57.1 | bc | 1.36 | a | 0.132 | bc | 0.412 | ab | 0.263 | ab | 0.122 | b |
| O19 | 51.4 | ab | 65.2 | ab | 1.27 | a | 0.163 | ab | 0.284 | d | 0.160 | bc | 0.138 | b |
| O19+PEG | 56.4 | a | 70.2 | a | 1.24 | a | 0.092 | c | 0.299 | cd | 0.094 | c | 0.150 | ab |
