# Supplementary material for: Root-Zone Warming Differently Benefits Mature and Newly Unfolded Leaves of Cucumis sativus L. Seedlings under Sub-Optimal Temperature Stress
Source: PLoS One. 2016 May 6;11(5):e0155298. doi: 10.1371/journal.pone.0155298 (PMC4859567; doi:10.1371/journal.pone.0155298)
Supplement: S2 Table — (DOCX) [file pone.0155298.s003.docx]

**S2 Table.** **The JIP-test results in the second true leaf of cucumber seedlings under different root-zone temperature and PEG treatments.** Means with different letters denote significant difference (*P* < 0.05, n = 3 or 4) by Tukey HSD.

| Treatment | PI_ABS_ | | RC/ABS | | TR_0_/ABS | | ET_0_/TR_0_ | | ET_0_/ABS | | RE_0_/ET_0_ | | RE_0_/ABS | | PI_total_ | |
| --- | --- | --- | --- | --- | --- | --- | --- | --- | --- | --- | --- | --- | --- | --- | --- | --- |
| S13 | 0.676 | ab | 0.236 | a | 0.737 | bc | 0.503 | b | 0.370 | b | 0.453 | b | 0.167 | b | 0.559 | bc |
| S13+PEG | 0.259 | c | 0.195 | bc | 0.679 | d | 0.378 | c | 0.257 | c | 0.383 | b | 0.099 | c | 0.167 | d |
| S19 | 0.582 | b | 0.164 | c | 0.720 | c | 0.578 | a | 0.416 | a | 0.559 | a | 0.232 | a | 0.738 | ab |
| S19+PEG | 0.650 | ab | 0.205 | ab | 0.737 | bc | 0.530 | b | 0.391 | ab | 0.411 | b | 0.161 | b | 0.455 | c |
| O19 | 0.792 | a | 0.195 | bc | 0.777 | a | 0.538 | ab | 0.417 | a | 0.556 | a | 0.232 | a | 0.998 | a |
| O19+PEG | 0.720 | ab | 0.222 | ab | 0.765 | ab | 0.497 | b | 0.380 | ab | 0.417 | b | 0.159 | b | 0.530 | bc |
